# Control Method of Cold and Hot Shock Test of Sensors in Medium

**DOI:** 10.3390/s23146536

**Published:** 2023-07-20

**Authors:** Jinming Tian, Yue Zeng, Linhai Ji, Huimin Zhu, Zu Guo

**Affiliations:** School of Electronic Engineering, Jiangsu Ocean University, Lianyungang 222000, China; tianjinming4213@126.com (J.T.); 2006000031@jou.edu.cn (L.J.); 2020210822@jou.edu.cn (H.Z.); 2022210605@jou.edu.cn (Z.G.)

**Keywords:** environmental test chamber, cold and hot shock test, K-means, support vector machine, neural network, sensor test

## Abstract

In order to meet the latest requirements for sensor quality test in the industry, the sample sensor needs to be placed in the medium for the cold and hot shock test. However, the existing environmental test chamber cannot effectively control the temperature of the sample in the medium. This paper designs a control method based on the support vector machine (SVM) classification algorithm and K-means clustering combined with neural network correction. When testing sensors in a medium, the clustering SVM classification algorithm is used to distribute the control voltage corresponding to temperature conditions. At the same time, the neural network is used to constantly correct the temperature to reduce overshoot during the temperature-holding phase. Eventually, overheating or overcooling of the basket space indirectly controls the rapid rise or decrease in the temperature of the sensor in the medium. The test results show that this method can effectively control the temperature of the sensor in the medium to reach the target temperature within 15 min and stabilize when the target temperature is between 145 °C and −40 °C. The steady-state error is less than 0.31 °C in the high-temperature area and less than 0.39 °C in the low-temperature area, which well solves the dilemma of the current cold and hot shock test.

## 1. Introduction

**Figure 1:**
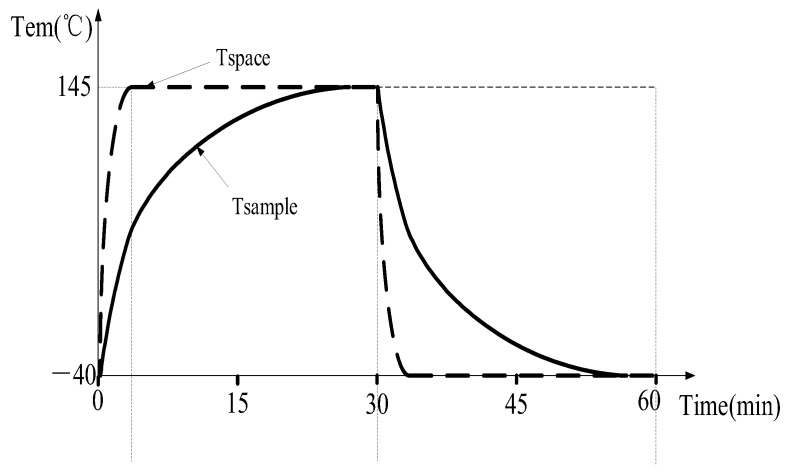
Existing control process ($T_{\mathrm{space}}$ is the temperature of the space inside the test chamber; $T_{\mathrm{sample}}$ is the temperature of the sample sensor).

Cold and hot shock test is a modern testing method that uses high- and low-temperature cycles to test the quality of sensors. In general, it can be tested directly in high- and low-temperature environment test chambers. The cold and hot shock test of the sensor in the medium is different from the direct cold and hot shock test. Due to the complexity and particularity of the medium, the sensor placed in it will lead to the characteristics of large inertia and time lag in the whole temperature control process. As shown in Figure 1, a complete hot and cold shock test is divided into high temperature for 30 min and low temperature for 30 min. The sample temperature needs to be kept at the target temperature for more than 15 min in each area. We chose the test standards of 145 °C and −40 °C, which are the most commonly used test standards for engine sensors. We can obviously find that the temperature of the chamber space increases rapidly in a very short time, but the temperature of the measured sensor cannot reach the target temperature in 30 min, showing a great lag. This phenomenon also occurs in low-temperature tests. The cold and hot shock test of the sensor in the medium using environmental test chamber is an important problem to be solved urgently in the current testing field. For example, the engine sensor needs to work in the oil medium, and the traditional hot and cold shock test often leads to problems with the produced engine sensor under long-term operation. Today, with the rapid development of intelligent driving, every sensor in the car may affect the safety of passengers. Therefore, we must simulate the working environment of the sensor more realistically. This effective solution not only improves the current domestic testing level but also reduces the production cost, ensuring better safety for product users.

As the control of cold and hot shock test of sensors in the medium is a new problem in the current testing field, there is no previous related research in the world. According to the analysis of its characteristics, its main purpose is to solve temperature control problems by lag, nonlinearity and large inertia. For different control objects, the temperature control scheme shows a strong diversity [1,2,3,4,5,6]. Zhou et al. [7] used the K-means clustering algorithm to cluster temperature measuring points in different locations for the optimal selection of machine tool temperature measuring points. The method effectively reduces the number of measuring points and improves testing efficiency, but there is a lack of effective automatic classification and judgment methods. Wang et al. [8] presented a GAPSO-optimized Fuzzy PID controller for electric-driven seeding. They combined GA and PSO to optimize fuzzy control, but the combination of the two methods is not clear, and their membership functions do not seem to be related to environmental changes. Liu et al. [9] used a neural network to adjust the PID parameters for solving the problem of temperature control system in a resistance furnace. However, it is difficult to obtain data set when using neural network in the whole system, and the environmental condition of resistance furnace is distinct.

In view of the advantages and disadvantages of the above temperature control problems and the particularity of the cold and hot shock test of the sensor caused by the medium environment [10,11,12], this paper combines the advantages of neural network and K-means clustering SVM classification method [13]. We propose to use K-means clustering SVM classification method as a whole, which can reflect the distribution of temperature difference within this medium environment and distribute different control signals. The neural network control is only adopted when the temperature difference between the medium temperature and the basket temperature is close. Because the range of temperature difference is small and the data available for adjustment are limited, it is convenient to sort out the artificial data set. The control effect of the network can effectively reduce overshoot and improve the stability of temperature control. The organic combination of these two designs can quickly and stably realize the control process for the cold and hot shock test of the sensor in the medium [14,15].

## 2. Test Requirements and Requirements Analysis

**Figure 2:**
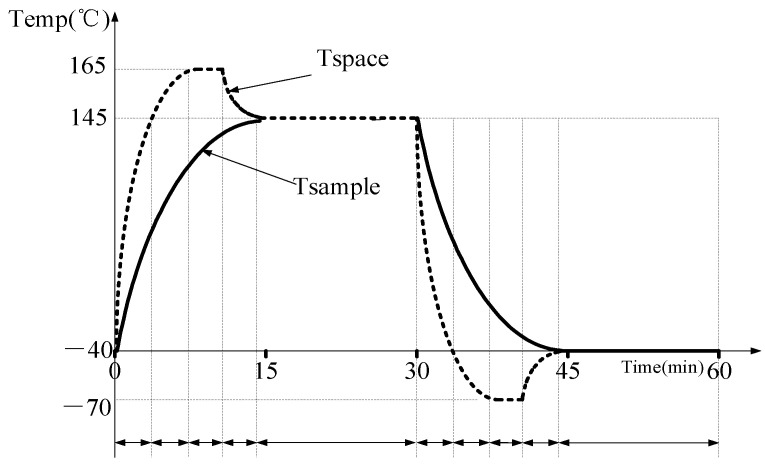
Ideal control process ($T_{\mathrm{space}}$ is the temperature of the space inside the test chamber; $T_{\mathrm{sample}}$ is the temperature of the sample sensor).

The cold and hot shock test of the sensor in the medium not only requires rapid heating or cooling to the target temperature within half an hour but also maintenance of the sensor at the target high or low temperature for more than 15 min to ensure effective testing. However, when the environmental test chamber itself is used to conduct a hot and cold shock test of the sensor in the medium, the temperature of the test chamber reaches the target temperature in a very short time; however, the sensor temperature struggles to reach the target temperature within the specified time. In order to meet the test requirements, when the target high temperature is 145 °C and the low temperature is −40 °C, the environmental test chamber is designed to quickly heat up to far more than 145 °C (Upper limit is 165 °C) in the high temperature area and then cool down. Similarly, reducing the temperature in the low temperature to a level much lower than −40 °C (with a lower limit of −70 °C) allows the temperature of the sensor in the medium to quickly reach and maintain the target temperature within a specified period of time. The ideal temperature curve is shown in Figure 2 below. Since it is not possible to maintain the actual situation at a constant target temperature, a maximum error of ±2 °C can be allowed depending on the circumstances.

### 2.1. Test Chamber Structure

**Figure 3:**
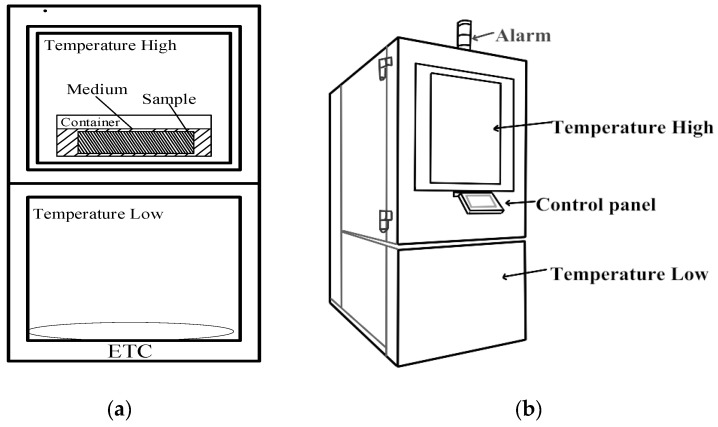
(**a**) Upright view structure of test chamber (ETC, Environment test chamber). (**b**) Three-dimensional diagram of structure.

The controlled test chamber is divided into two layers: the upper layer is the hot area, and the lower layer is the cold area. As depicted in Figure 3a, the sample sensor is positioned within the medium and enclosed in a box. The internal platform of the environmental test chamber will automatically elevate and descend to transport the sample between the high-temperature and low-temperature boxes when necessary. This movement usually occurs every 30 min. As shown in Figure 3b, the rising and cooling process is controlled by the control panel of the test chamber. When the temperature exceeds the maximum limit or encounters other special circumstances, the test chamber will stop running, and the alarm light above it will beep simultaneously. The controlled voltage reflects the temperature experienced within the test chamber. Take the hot area as an example. When the received voltage signal is lower than the set value, the temperature of the test chamber is increased. On the contrary, when its value is above the set point, the test chamber stops heating up.

### 2.2. Principle of Control Process

Because the power of the environmental test chamber cannot be directly controlled, the heat conduction characteristic of the temperature is used to control the basket temperature from being overheated or overcooled. This property of temperature indirectly affects the rate at which the sample temperature rises and falls. Take the heating up process a s an example. According to the general idea, we should first control the basket temperature to increase to the limit temperature with the maximum power. Then, we should stop heating at a specific node. Follow these steps to reduce the temperature of the basket to the target temperature. In the temperature holding stage, the temperature of the basket is controlled to increase when the sample temperature is lower than the target temperature. Once the temperature exceeds the target temperature, the basket is controlled to stop heating. The internal temperature field is uniform by using the heat dissipation fins inside and outside the container. The testing temperature can be quickly reached within the allotted time and maintained at a stable level. Finally, the sample temperature should exhibit a curve with minimal fluctuations around the target temperature. Take the process of high-temperature area as an example, the following is a step-by-step process. The process in the low-temperature area is similar.

(1) Space temperature rises rapidly; the sample temperature increases accordingly.
(2) Space temperature reaches the high-temperature limit and maintains; the sample temperature continues to rise.
(3) Space temperature gradually drops to the target temperature, while the sample temperature gradually increases to the target temperature.
(4) Space temperature oscillates around the target temperature, while the sample temperature remains stable at the target temperature.

**Figure 4:**
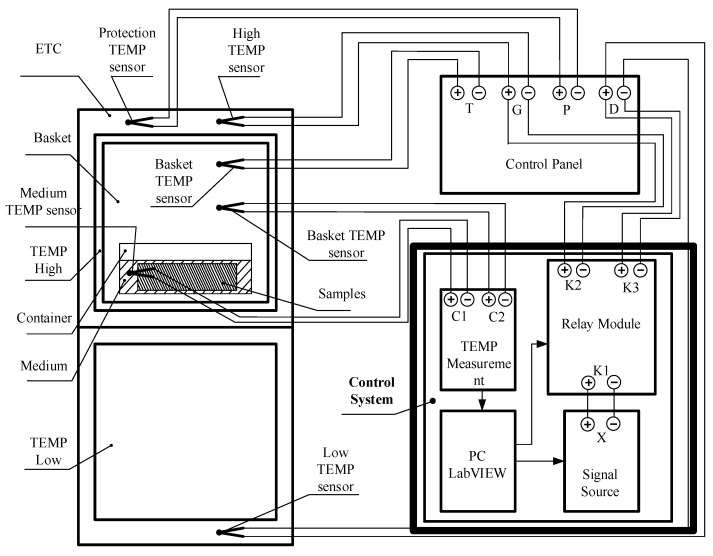
Connection mode of the system and chamber.

This paper aims to adjust the voltage output to the control panel of the test chamber based on the temperature information from two groups of sensors input into the temperature control system [16]. As shown in Figure 4, the output signal of the control system is used to simulate the temperature signal received by the test chamber. Finally, the temperature of the medium and sensor is controlled and stabilized at the target value by the test chamber. In general, the output voltage of our control system only affects the control panel. These control voltages represent the values displayed on the control panel. When these values are significantly lower than the target temperature, the heating power of the test chamber is higher. When these values are close to the target temperature, the test chamber has less power. Once the actual temperature of the space reaches the upper limit, the system will automatically stop heating.

## 3. Control Method

According to the design logic, if there is a significant temperature difference between the sensor in the medium and the temperature in the basket, the rate of temperature rise and drop should be accelerated. When the temperature difference is small, the increase or decrease in temperature should be minimized or halted. However, it is impossible to select the appropriate node adjustment based solely on experience. Furthermore, there is no accurate expert guidance available to make the choice [17,18]. If a continuous and nonlinear method, such as a neural network, is used to adjust each output voltage, we find that these adjustments are unnecessary during the stage of drastic temperature changes. For example, when the temperature is at that level, the difference between 2 V and 2.5 V is barely noticeable on the temperature curve. We studied the cause of this phenomenon and finally found that when the output voltage values represent lower display values, it means that these values are far from the target temperature. Currently, the control system of the test chamber itself does not deem it necessary to adjust the power. Another important reason is that when the temperature difference is large enough, the small voltage change is insufficient to significantly increase the slope of the curve. Therefore, in order to divide the temperature difference data more effectively and clearly, the clustering algorithm is selected to separate the data into rising and cooling processes every time the medium condition changes. According to the characteristics of the input data for this system: the sample complexity is low, the human experience is clear, and the data set has minimal noise. The K-means cluster analysis method [19] is chosen to analyze the cold and hot shock tests data of the test chamber. The appropriate evaluation scheme is selected to obtain the optimal data classification scheme. Based on the clustering results, the support vector machine (SVM) [20] is used to classify and assign the control voltage to each classification. If the neural network is used to solve the classification problem, the data set is still based on the SVM classification. Adopting the method of neural network is obviously superfluous. The value of the control voltage is determined by the self-control system of the test chamber.

### 3.1. K-Means Clustering

K-means is a classical and efficient unsupervised learning method that can divide the sample set into K clusters based on the distance between the sample data. In this way, the points within the cluster are connected as closely as possible, while maximizing the distance between clusters. It is characterized by high cohesion and low coupling. When expressed by the formula, the cluster is divided into ($C_{1}$,$C_{2}$,...$C_{k}$). Currently, we need to find K centroids to minimize the squared error E. The smaller E is, the higher the similarity within the same cluster. The expression of E is shown in Equation (1).

(1) $$E=\sum_{i=1}^{k}\sum_{x\in C_{i}}||x-\mu_{i}|{|}_{2}^{2}$$

$\mu_{i}$ is the mean vector expression of the cluster $C_{i}$, as shown in Formula (2), represents the centroid as well.

(2) $$\mu_{i}=\frac{1}{\left|C_{i}\right|}\sum_{x\in C_{i}}x$$

Since K initial centroids need to be selected at the beginning of the algorithm, the selection of initial centroids in the K-means-based clustering method is completely random. If each clustering result is based on a different centroid, it becomes impossible to evaluate whether the centroid or the change of K value affects the clustering effect after the change in K value. So, it is necessary to obtain the centroid using the same method in order to compare the effect of different K values. The K-means++ algorithm is used to optimize the random initialization of centroids. The main idea of K-means++ [21] clustering is to select a randomly chosen point $\mu_{1}$ from the input dataset as the initial clustering center. For each point $x_{i}$ in the dataset:

(1) Calculate the distance between it and the nearest clustering center in the selected cluster center, as shown in Formula (3).
(2) Select a new data point as the new clustering center, and the point with a larger D(x) value is more likely to be chosen as the clustering center.
(3) Repeat the above steps until K cluster centroids are selected.
  (3) $$D\left(x_{i}\right)\;=arg\;min||x_{i}-\mu_{j}|{|}_{2}^{2},\;j=K_{selected}\;$$

**Figure 5:**
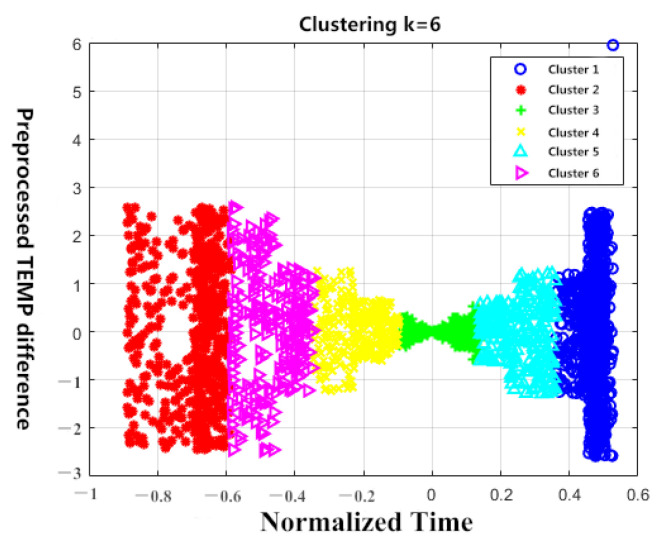
Clustering results of K = 6.

**Figure 6:**
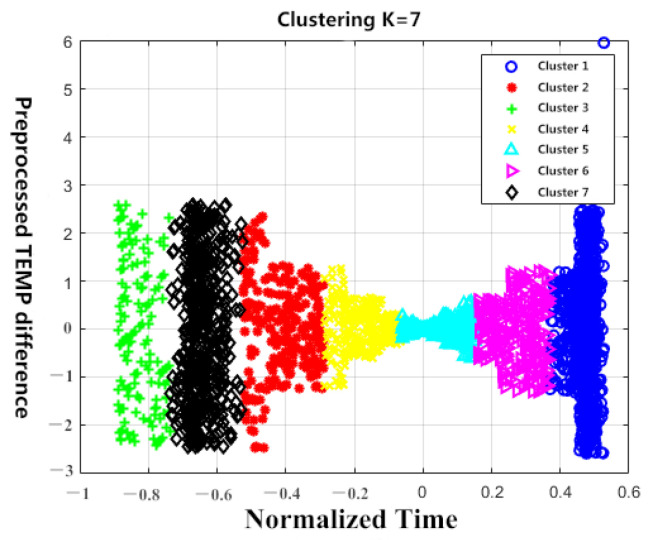
Clustering results of K = 7.

These K centroids are used as initialization centroids for the K-means algorithm. A set of rising and cooling data with a target high temperature of 145 °C and a low-temperature of −40 °C is selected for cluster analysis. The cases of K = 6 and K = 7 are shown in Figure 5 and Figure 6 below. Because the temperature difference data is one-dimensional, it is represented as a line on the graph. Therefore, in order to improve clustering and visualization, the two-end temperature difference data is used to enhance the dimension. After performing PCA, normalization, and dimensionality reduction, the following scatter plot is generated.

The Gap Statistic (GS) method [22] is used to evaluate the clustering results for different K values and select the best clustering results. The GS method was first proposed by Tibshirani to solve the problem of determining the optimal number of clusters. The main idea is to describe the difference between the observed value of the sample and the expected value under a reference distribution using gap statistics. Obtain the Euclidean distance between the sample points in Formula (4) and the expected value under a uniform distribution in Formula (5) to determine the optimal maximum K value.

{(4)Dk=2nk∑X𝒾∈CkX𝒾−μk2 (5)GapK=E(logDk)−logDk 

**Figure 7:**
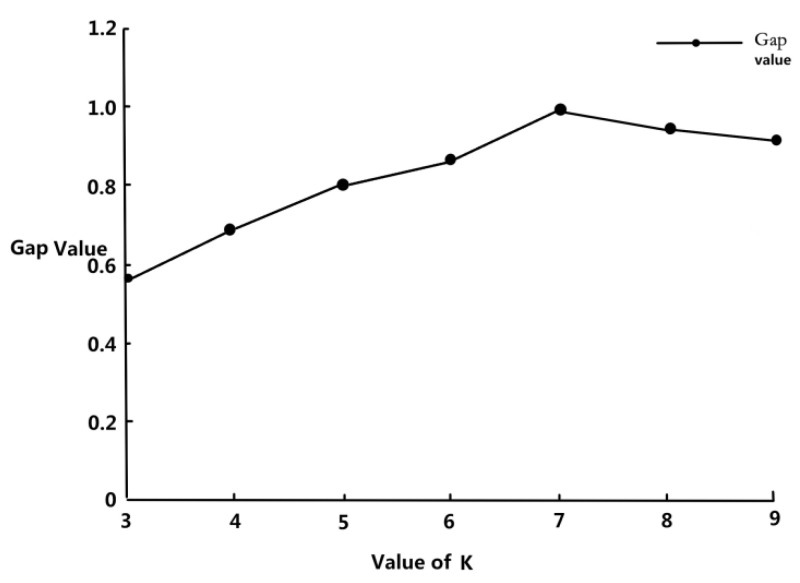
Values of Gap in cluster K from 3 to 9.

Because the Gap value does not have control significance when the number of clusters is less than or equal to two, it is calculated for more than three clusters. As shown in Figure 7 below, K = 7 has the highest Gap value, indicating that seven is selected as the optimal clustering result.

### 3.2. SVM Control Voltage Classification

After completing the clustering of the data and selecting the optimal clustering scheme, it is also necessary to effectively differentiate between each cluster in order to achieve a more accurate mapping between input and output. At the same time, since the small voltage difference cannot significantly affect the slope of the heating curve, we can arrange three different voltages during the heating stage based on the clustering results. Therefore, controlling the cold and hot shock test of the sensor in the medium can be considered a classification problem. The entire process can be controlled by classifying different voltage values based on varying temperature differences. Support Vector Machine (SVM) is a highly effective tool for addressing small sample, nonlinear classification problems. Because the temperature difference data is linearly separable, it is easier to use the SVM method to classify the clustering results. Some points do not appear to be linearly segmented when extended to two-dimensional space, but the nature of the original data is linearly separable. So, we can still simplify the problem to a linearly separable case without adding nonlinear kernel functions. The core idea of SVM is to find a hyperplane that can separate different types of data with maximum intervals, thereby achieving the purpose of classification [23]. In the case of linear inseparability, the kernel function can also be introduced to transform the problem into a linear classification problem in a high-dimensional feature space. Because the processed temperature difference data is approximately linearly separable, the problem of finding the maximum hyperplane can be directly transformed into a convex quadratic programming problem with inequality constraints. As shown in Formula (6), where W is the weight vector, X is the training dataset, and *b* is the scalar.

(6) $$W^{T}X+b=0$$

By solving the quadratic programming problem, the values of W and *b* can be obtained as shown in Equation (7). The corresponding class mark $X^{𝒾}$ value $y_{𝒿}$ is 1 or −1, which represents the positive or negative of the classification. It can also be expressed in the form of Lagrangian duality, as shown in Formula (8).

(7) $$\left\{\begin{array}{c} \min\;\frac{1}{2}{\left|\left|W\right|\right|}^{2}\\ s.t.1-y_{𝒿}\left(WX^{𝒾}+b\right)\leq 0,𝒾=\left\{1,2,3\ldots n\right\} \end{array}\right.$$

(8) $$F\left(X\right)=\sum_{𝒾=1}^{n}\alpha_{𝒾}y_{𝒾}{𝓍}_{𝒾}^{T}X+b$$

In this paper, the training and prediction process of the SVM model is implemented using the LIBSVM toolbox in Matlab. Because it is a linearly separable case, the penalty factor C is set to 0, and the kernel function adopts a linear kernel as shown in Formula (9).

(9) $$K\left(X,Z\right)=X\cdot Z$$

**Figure 8:**
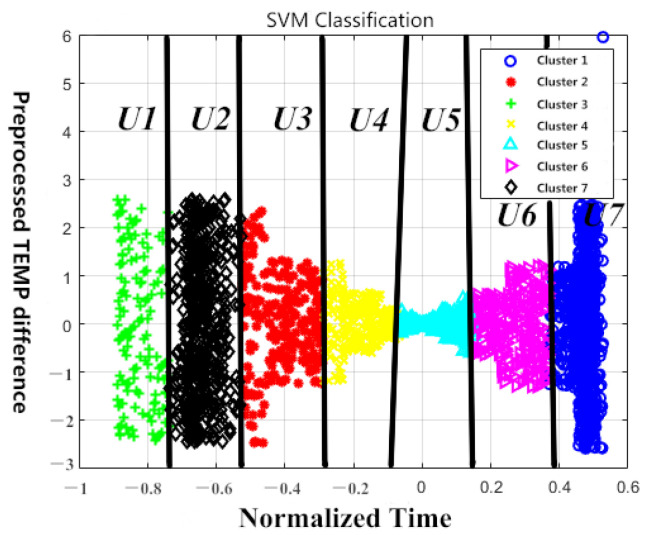
SVM Classifications at K = 7 (*U*1 to *U*7 are the control voltages).

SVM was originally designed to solve the two-classification problems. In order to solve this multi-classification problem, we need to design and use multiple classifiers for classification [24]. The commonly used multi-classification schemes are the classical one-to-many and one-to-one schemes. The one-to-many (one-against-all) method takes one category as the positive class and the other categories as the negative class. The classifier is then constructed in turn, and finally, the samples are classified into multiple categories. On the other hand, the one-to-one (one-against-one) approach requires the construction of k (k − 1)/2 classifiers [25]. However, the accuracy does not show significant improvement in the case of existing clustering. Therefore, one-to-many scheme is adopted for designing the classifier. The visualization of the case classification when selecting K = 7 is shown in Figure 8 below. 

A positive voltage value indicates that the sample is in a high-temperature area, while a negative voltage value indicates that the sample is in a low-temperature area. The switching between them depends on the relay module shown in Figure 4. The distribution voltage of each stage is determined based on the temperature difference data, as shown in Table 1 below.

**Figure 9:**
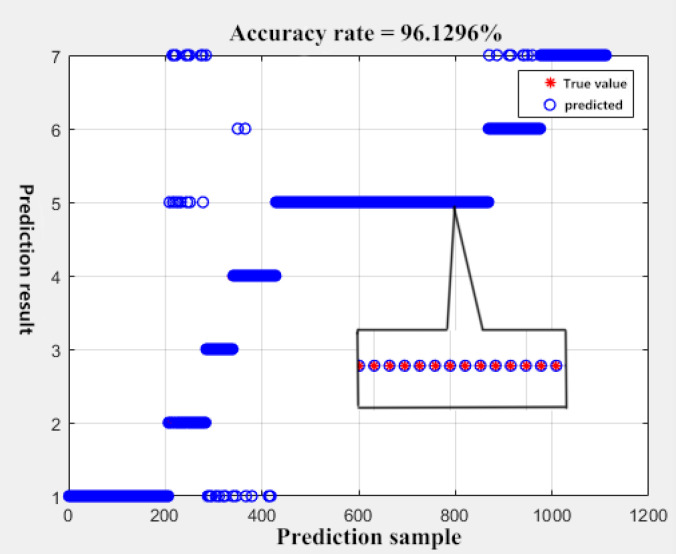
SVM classification prediction.

Select 2593 sets of data as the training set and 1109 groups as the test set. When dividing the test set data for regression testing of SVM’s 7 classification [26], the accuracy is 96.1296% after 500 iterations. As shown in Figure 9 below, because the classification cannot completely separate each cluster, there are some outliers. The presence of these outliers can lead to abrupt fluctuations in the output voltage, which is detrimental to the control system’s reliability and can result in hardware damage. Therefore, the Kalman filter module is added in the software design to filter the signal and produce an output. This effectively prevents sudden changes in the signal [27]. The positive voltage value cannot appear in the low-temperature area, and the negative voltage value cannot appear in the high-temperature area either. Otherwise, it will cause test interruption and trigger the equipment’s alarm. Therefore, in order to ensure the long-term stable operation of the equipment, a voltage limiting filter is designed at the output to ensure that each output voltage complies with the current equipment’s specifications.

### 3.3. Back Propagation Neural Network

The control effect of using only the SVM classification method based on cluster analysis essentially fulfills the condition of not exceeding an overshoot of ±2 °C in the test environment. However, it is easy to exceed the limit when the medium environment changes. In order to ensure that the system meets the test requirements within a specific range of environmental changes, it is necessary to minimize the overshoot as much as possible.

In the actual testing process, it has been observed that during the temperature holding stage, following a simple logic of heating up when the temperature is lower than the target temperature value and stopping heating when it is higher than the target temperature value results in a significant overshoot because the medium causes a large inertia of heat transfer in the system. The fine-tuning process of adjusting temperature, whether increasing or decreasing, has a significant delay. Therefore, simply dividing the temperature holding stage into linear divisions is insufficient to achieve optimal control. At this stage, unlike the previous stage with a large temperature difference, the rate of temperature difference change in the container space remains stable at a small value below 0.1 °C/s. The change in the control voltage can effectively influence the temperature change rate of the sample in order to solve this problem, considering the strong fitting ability of neural network for nonlinear functions. Consider the Back Propagation (BP) neural network, specifically the back propagation neural network, to solve the control problem during the temperature holding phase [28]. 

**Figure 10:**
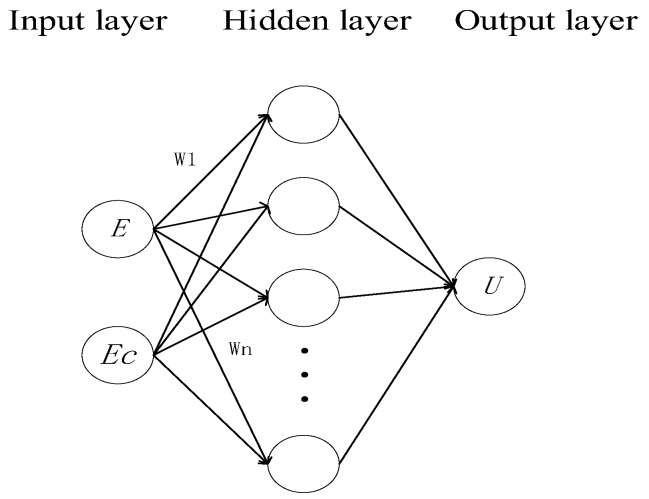
BP neural network structure.

BP neural network is a classical feedforward neural network that uses a backpropagation method to update weights. It includes an input layer, a hidden layer, and an output layer. The neurons between layers adopt a fully connected structure, which propagate forward using randomly initialized weights. The network backpropagates and updates weights according to the difference between the output value and the expected value in order to achieve self-learning. In theory, a neural network can approximate any nonlinear function. Therefore, by training the network’s parameters with high-quality data sets, it can effectively solve the problem of temperature retention phase. The structure of the BP neural network is shown in Figure 10 below. The target high temperature is 145 °C and the low temperature is −40 °C. The input layer *E* is calculated as the difference between temperature signal *T_S_* from the basket sensor and the temperature signal *Tc* from the sensor. Additionally, *E_C_* represents the temperature difference change rate, as shown in Formula (10).

(10) $$\left\{\begin{array}{c} E=T_{s}-T_{c}\\ E_{C}=\frac{dE}{dt} \end{array}\right.$$

*E_C_* contains more comprehensive information, and even if the medium environment changes greatly, we can receive timely feedback through *E_C_*. If only *T_S_* and *T_C_* are used as input variables, the effect of the system cannot be maintained when the target temperature and medium environment change. The model consists of a single hidden layer, and the output is represented by voltage U. A full connection structure is adopted between layers, and the weight updating mode is backpropagation [29].

The data set used in the training is modified based on the initial data generated using SVM classification control. The key point of overshoot occurs just after the rising and cooling phase. The basket temperature always fluctuates, ranging from temperatures much higher than the target temperature (in the high-temperature area) to temperatures much lower than the target temperature (in the low-temperature area). Therefore, the mapping of input and output data can be adjusted approximately based on the overshoot. According to the actual test, the basket needs to be cooled by 5 °C beforehand if the high-temperature area is adjusted by more than 2 °C. If there is a 2 °C overshoot in the low-temperature area, it is necessary to increase the temperature of the basket by 10 °C ahead of schedule. Based on the above experience, when the target temperature is 145 °C and −40 °C, thresholds of 140 °C and −30 °C are set to switch between SVM classification control mode and neural network control mode, respectively.

Because the need for neural network accuracy is higher than the prediction ability, more hidden layer neurons are selected. At the same time, it is necessary to maintain effective control over the medium environment while allowing for a certain degree of change. Therefore, the network should retain a certain level of generalization ability. The mean square error (MSE) in Formula (11) and the mean absolute error (MAE) in Formula (12) are used to measure the performance of the network [30]. MSE measures the accuracy of the output results. The smaller the MSE, the smaller the error [31,32]. MAE only measures the average magnitude of the prediction error.

(11) $$\mathrm{MSE}=\frac{1}{m}\sum_{i=1}^{m}{\left(y_{\mathrm{test}}^{\left(i\right)}-{\hat{y}}_{\mathrm{test}}^{\left(i\right)}\right)}^{2}$$

(12) $$\mathrm{MAE}=\frac{1}{m}\sum_{i=1}^{m}|y_{\mathrm{test}}^{\left(i\right)}-{\hat{y}}_{\mathrm{test}}^{\left(i\right)}|$$

**Figure 11:**
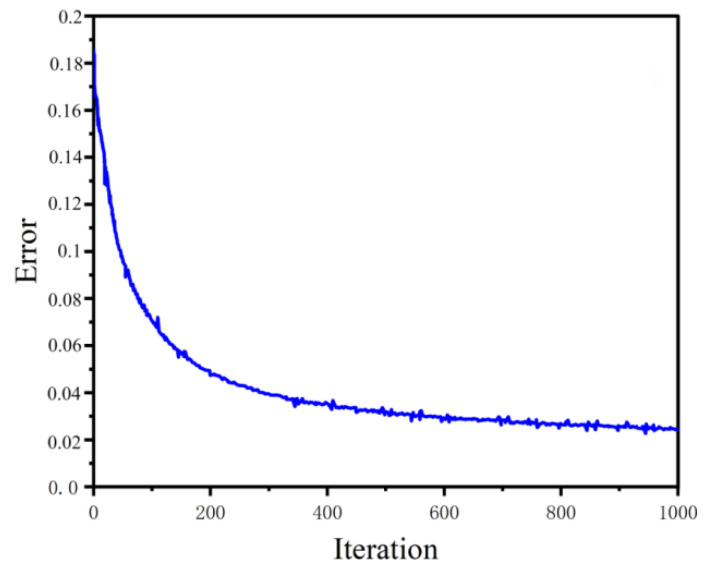
Error with iterations.

After 1000 iterations, the learning rate is 0.02, and there are 1136 sets of input and output data for training. Use Mean Squared Error (MSE) as the loss function. The error decreases as the number of iterations increases, as shown in Figure 11. 

Selecting 300 groups as the test set, the performance evaluation indicators are shown in Table 2 below. The predicted output voltage is essentially consistent with the actual voltage.

In order to show the process of neural network adjustment more clearly, several pieces of data were extracted from continuous data sets, as shown in Table 3 below. In the table, the target high temperature is 145 °C and the low temperature is −40 °C.

### 3.4. Overview of Control Methods

To better demonstrate the application of these methods, step-by-step explanations are provided below.

(1) Obtain raw data sets based on experience and experiments.
(2) Use a clustering algorithm to evaluate and obtain the optimal clustering after data preprocessing.
(3) Classify the output voltage using SVM and determine the corresponding voltage value through experimentation.
(4) Accurately adjust the data of temperature holding phase on the basis of existing data. Use this data to train the neural network.
(5) Conduct experiments and set up appropriate nodes to effectively integrate the two methods. Repeat experiments to verify the stability and effectiveness of the method.

### 3.5. Temperature Compensation

Due to the change of ambient temperature from −50 °C to 170 °C, the characteristics of the sensitive elements in the Pt100 temperature sensor vary with temperature. However, the resistance and capacitance of the signal processing circuit are not significantly affected. If the transmitted signal is not processed, it will affect the control accuracy [33,34]. It is difficult to meet the precision requirements simply by adding hardware compensation to the sensor. The temperature compensation module is used to compensate for temperature variations in the temperature control system. In the software design, the temperature compensation value primarily affects the input signal. The algorithm’s input will change accordingly with the change in the compensation value, thus affecting the output voltage value.

After conducting actual debugging, it was found that the influence of the compensation value on the system follows a normal distribution, with the maximum value occurring at −2. It is designed as an external adjustable parameter that allows testers to modify the compensation value during the actual test.

## 4. Experiments

**Figure 12:**
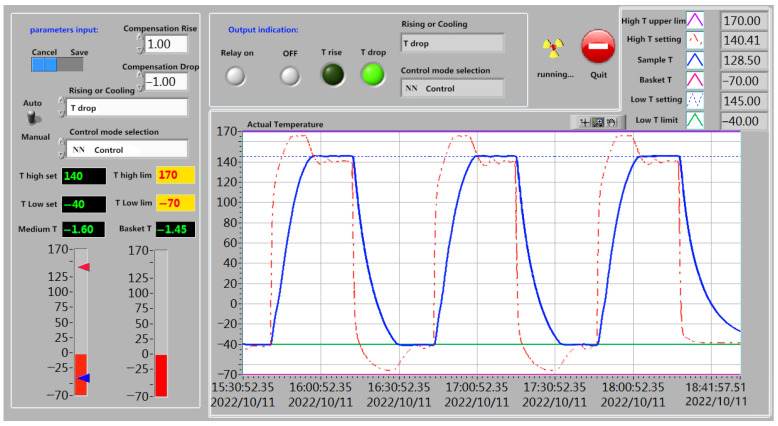
System main interface.

The current running status panel of the system is shown in Figure 12. The main part of the program is written using LabVIEW software [35,36]. The design of the front panel is displayed with a graphical interface to facilitate the operation and adjustment of field personnel. After clicking to run the system, the test parameter setting interface will pop up first. Secondly, fill in the upper and lower limits of the high and low temperatures according to the test requirements. Thirdly, set the temperature and temperature compensation. Finally, click the OK button to exit the parameter setting interface and enter the main interface of the control system.

**Figure 13:**
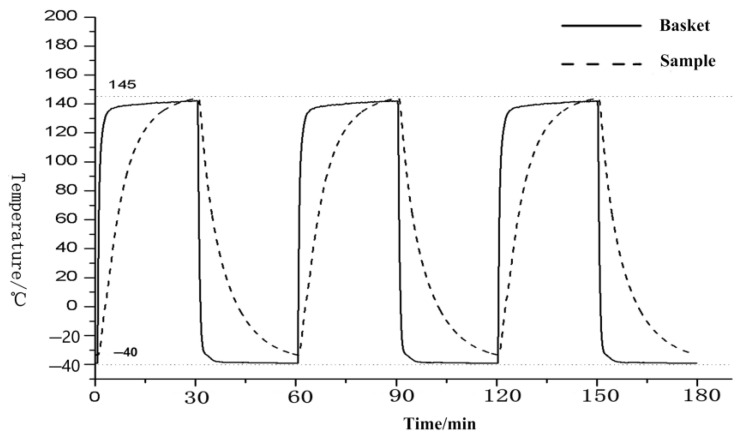
Automatic Control of Environmental Test Chamber.

Set the target high temperature to 145 °C and the target low temperature to −40 °C for system testing. At the beginning of the test, two containers with a fixed position were used, and the internal volume of each container was filled with 300 mL of oil medium liquid. As shown in Figure 13, without any external intervention in the environmental test chamber, the sample temperature gradually increases for 28 min to reach the target high temperature of 145 °C. However, the target low temperature of −40 °C cannot even be achieved at all. The test results fall far short of the required temperature maintenance at the target temperature for 15 min.

**Figure 14:**
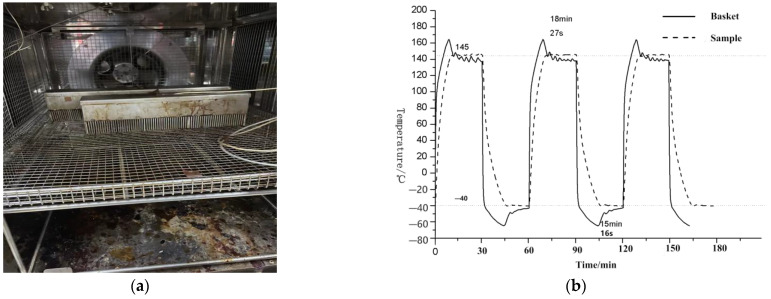
(**a**) The internal arrangement of the basket. (**b**) Corresponding control temperature curve.

After implementing the SVM control method, which is based on K-means and involves properly adjusting the control voltage, the container holding the medium is positioned as depicted in Figure 14a. When starting the test, the basket temperature increases rapidly to 165 °C. It is important to note that for the long-term use of the equipment, the high temperature should not exceed 165 °C at the high end and should not go below −70 °C at the low end. This temperature range has an impact on the rapid increase in the sample temperature. After reaching a certain point, the basket temperature starts to decrease. Finally, the temperature of both areas is around 145 °C, and the low-temperature area is also maintained at −40 °C. 

However, primarily due to the disparity in heat transfer rates between low and high temperatures, the control accuracy and speed in low temperature are lower than those in high temperature. As shown in Figure 14b, the target holding time at high temperature is 18 min and 27 s, while at low temperature it is 15 min and 16 s, which meets the test requirements satisfactorily.

**Figure 15:**
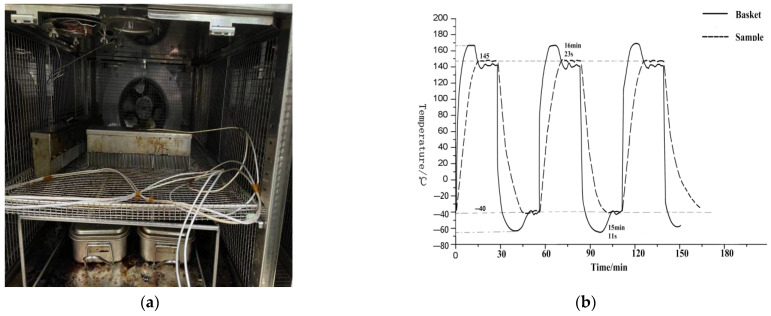
(**a**) The internal arrangement of the basket. (**b**) Corresponding control temperature curve.

After repositioning the container, two additional boxes of medium liquid, filled with oil and with a capacity of 200 mL, are added to the basket. As shown in Figure 15a below, the heating rate of the medium is affected by changes in position and medium mass. Therefore, it can be observed from the rising and falling curve that the holding time of the basket temperature in the high position increases significantly. As shown in Figure 15b, the target holding time in the high-temperature area is 16 min and 23 s, while in the low-temperature area is 15 min and 11 s. However, the effect is slightly lower than what is depicted in the above figure, which can be attributed to the upper limit of equipment performance. The control effect still meets the testing requirements.

**Figure 16:**
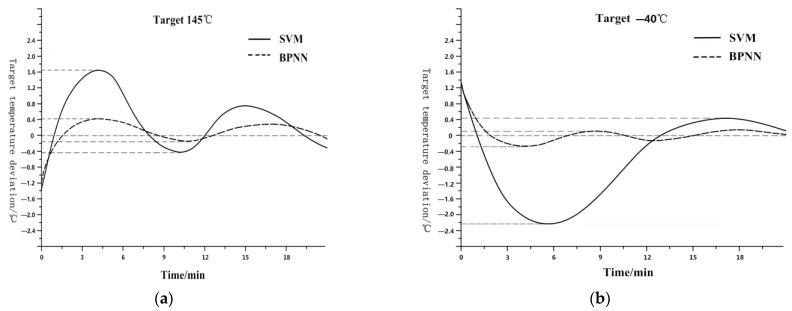
(**a**) Comparison in High-Temperature area. (**b**) Comparison in Low-Temperature area.

Although the test requirement is to control the temperature in the medium at ±2 °C, the SVM control using K-means has essentially met the control requirements. However, in fact, as mentioned above, the uncertain factors of the media cannot be avoided. For example, it is impossible for the tester to consistently position the container in the same place every time, and it is not possible to strictly guarantee that the amount or even the type of medium in the container will be consistent with the experimental environment. Because SVM cannot adjust the output voltage value in detail, the system overshoot is larger, making it easier to exceed the test requirements. It is necessary to minimize the overshoot. After controlling the temperature holding stage of the display state of Figure 14 using a BP neural network, the effect on the high-temperature area is shown in Figure 16a. The overshoot is reduced from 1.65 °C to 0.4 °C. Similarly, in the low-temperature area, the overshoot is reduced from 2.2 °C to 0.3 °C, as shown in Figure 16b. Whether at high or low temperatures, the stability of the system has been significantly improved.

Because the sample needs to undergo cyclic impact testing in the medium to assess its quality during cold and hot shock tests, 10 complete impact testing processes are selected. The data for the high-temperature area is presented in Table 4, while the data for the low-temperature area is presented in Table 5. Upon analyzing the data in the tables, it is evident that the steady-state error in the high-temperature region is below 0.31 °C, and in the low-temperature area, it is below 0.39 °C. These results indicate that the cold and hot shock test requirements are satisfactorily met.

## 5. Conclusions

(1) This article proposes a control method that combines K-means and SVM with neural network correction. The method is designed to achieve the efficient control of the cold and hot shock testing of the sensor in the medium. On the whole, the system utilizes K-means clustering analysis of temperature data and the SVM method to distribute the control voltage signal. In detail, the output control voltage is fine-tuned by combining it with neural network control during the temperature holding stage. Timely control the temperature changes in the basket space in order to indirectly regulate the temperature of the sample sensors in the medium. With the combination of overall control and detail control, this method is highly effective in ensuring that the sample’s temperature in the medium meets the industry’s test requirements during the cold and hot shock testing.
(2) Combined with the graphical interface of LabVIEW, the entire system effectively enhances the stability and controllability of the current cold and hot shock test. It realizes the effective control of the sensor’s temperature in the medium, as well as the display and recording of the test data.

However, the controller does not take all variable factors into account as input and can only maintain the control effect within a certain range of medium. When the environment of the medium changes too much, the control effect will be affected. In the future, the amount and types of more medium will be expanded as the input to the system. Several commonly used test indicators will be integrated on the basis of the existing data to achieve targeted control of a wider range of test environments.

## Figures and Tables

**Table 1:** Voltage in the temperature difference range.

TEMP difference/°C	136.631–87.167	87.167–53.449	53.449–15.902	15.902–−4.463	−4.463–−31.357	−31.357–−66.852	−66.852–−140.74
U/v	0.6	1.5	4.2	6.1	5.9	−0.6	−2

**Table 2:** Performance of Neural Network in test sets.

Neural Network	MSE	Maximum Error/%	MAE
Evaluation value	0.038	4.193	0.158

**Table 3:** Typical data extracted from a neural network control process.

Ts/°C	Tc/°C	E/°C	Ec (°C/s)	U (True)/v	U (Prediction)/v
138.844	144.976	−6.132	0.08	5.72	5.56
139.003	144.957	−5.953	0.08	5.64	5.45
141.024	144.986	−3.962	0.05	5.85	5.95
141.074	144.996	−3.922	0.04	5.86	5.97
141.174	145.0168	−3.842	0.05	6.12	6.29
141.213	145.0367	−3.822	0.02	6.12	6.33
141.253	145.0397	−3.785	0.03	6.15	6.3
141.326	145.062	−3.736	0.02	6.22	6.47
−42.081	−39.974	−2.107	0.04	−6.22	−6.25
−42.031	−39.984	−2.047	0.03	−6.19	−6.31
−41.991	−40.004	−1.987	0.03	−6.11	−6.24
−41.683	−40.113	−1.569	0.02	−6.27	−6.5
−41.593	−40.153	−1.440	0.01	−6.31	−6.37
−41.474	−40.173	−1.300	0.02	−6.23	−6.44
−41.356	−39.946	−1.409	0.03	−5.85	−5.68
−41.322	−39.923	−1.398	0.01	−5.82	−5.59

**Table 4:** 10 consecutive cold and hot shock tests (high-temperature area).

Number	Overshoot/°C	Steady Time/min	Steady State Value/°C
1	0.37	16.33	145.11
2	0.3	16.25	145.24
3	0.22	15.87	145.13
4	0.41	16.08	145.31
5	0.38	16.22	145.22
6	0.23	15.61	144.91
7	0.27	15.74	145.15
8	0.36	16.06	144.85
9	0.26	16.21	145.09
10	0.25	16.15	145.14

**Table 5:** 10 consecutive cold and hot shock tests (low-temperature area).

Number	Overshoot/°C	Steady Time/min	Steady State Value/°C
1	0.28	15.12	−40.25
2	0.41	15.08	−40.31
3	0.27	15.19	−40.11
4	0.51	15.22	−40.23
5	0.36	15.11	−40.18
6	0.32	15.42	−40.25
7	0.64	15.03	−40.39
8	0.25	15.25	−40.14
9	0.19	15.06	−39.94
10	0.35	15.33	−40.17

## Data Availability

Not applicable.
